# RETRACTION: Thujone‐Rich Fraction of *Thuja occidentalis* Demonstrates Major Anti‐Cancer Potentials: Evidences from In Vitro Studies on A375 Cells

**DOI:** 10.1155/ecam/9814357

**Published:** 2026-03-16

**Authors:** Evidence-Based Complementary and Alternative Medicine

RETRACTION: R. Biswas, S. K. Mandal, S. Dutta, S. S. Bhattacharyya, N. Boujedaini, A. R. Khuda‐Bukhsh, “Thujone‐Rich Fraction of *Thuja occidentalis* Demonstrates Major Anti‐Cancer Potentials: Evidences from In Vitro Studies on A375 Cells,” *Evidence-Based Complementary and Alternative Medicine*, 2011, 568148, https://doi.org/10.1093/ecam/neq042.

The above article, published online on 20 February 2011 in Wiley Online Library (https://wileyonlinelibrary.com), has been retracted by agreement between the editorial board and John Wiley & Sons Ltd. The retraction has been agreed due to multiple concerns identified with the figures. As originally raised on PubPeer [1], there are concerns with Figures 10, 12, and 16. On subsequent investigation, additional concerns were also identified with Figure 17. A summary of the concerns is as follows:

• In Figure 10,
  ◦ Panels D and E contain overlapping sections in the bottom and top part of the panels, respectively.
  ◦ Panel F is identical to panel H.
• In Figure 12, image analysis shows similarities and overlapping data points between plots A and B.
• In Figure 16,
  ◦ Panels E and H contain overlapping sections in the bottom and top part of the panels, respectively.
  ◦ Panel F is identical to panel G.
• In Figure 17,
  ◦ The second and fourth Bax bands appear identical
  ◦ The second and fourth ß‐actin bands appear similar
  ◦ The second and fourth Caspase‐3 bands appear similar
  ◦ The second and fourth Cytochrome C bands appear similar
  ◦ Image analysis has revealed evidence of undeclared splicing

We asked the authors for their explanation, and they did not agree that the images were overlapping. As their response did not resolve our concerns, the Editorial Board recommended the retraction of the article.

The authors did not respond to indicate their agreement to the retraction.
